# Characterization of Skeletal Muscle Biopsy and Derived Myoblasts in a Patient Carrying Arg14del Mutation in *Phospholamban* Gene

**DOI:** 10.3390/cells12101405

**Published:** 2023-05-17

**Authors:** Simona Zanotti, Michela Ripolone, Laura Napoli, Daniele Velardo, Sabrina Salani, Patrizia Ciscato, Silvia Priori, Deni Kukavica, Andrea Mazzanti, Luca Diamanti, Elisa Vegezzi, Maurizio Moggio, Stefania Corti, Giacomo Comi, Monica Sciacco

**Affiliations:** 1 Neuromuscular and Rare Disease Unit, Fondazione IRCCS Ca’ Granda Ospedale Maggiore Policlinico, Via Francesco Sforza 35, 20122 Milan, Italy; 2 Neurology Unit, Fondazione IRCCS Ca’ Granda Ospedale Maggiore Policlinico, 20122 Milan, Italy; 3 Department of Molecular Medicine, University of Pavia, 27100 Pavia, Italy; 4 Department of Molecular Cardiology, IRCCS ICS Maugeri, 27100 Pavia, Italy; 5 Laboratory of Molecular Cardiology, Centro Nacional de Investigaciones Cardiovasculares Carlos III, 28029 Madrid, Spain; 6 Neuroncology Unit, IRCCS Mondino Foundation, 27100 Pavia, Italy; 7 Department of Brain and Behavioral Sciences, University of Pavia, 27100 Pavia, Italy; 8 IRCCS Mondino Foundation, 27100 Pavia, Italy; 9 Dino Ferrari Centre, Department of Pathophysiology and Transplantation (DEPT), University of Milan, 20122 Milan, Italy

**Keywords:** phospholamban, Arg14del, skeletal muscle, aggresomes

## Abstract

Phospholamban is involved in the regulation of the activity and storage of calcium in cardiac muscle. Several mutations have been identified in the *PLN* gene causing cardiac disease associated with arrhythmogenic and dilated cardiomyopathy. The patho-mechanism underlying *PLN* mutations is not fully understood and a specific therapy is not yet available. *PLN* mutated patients have been deeply investigated in cardiac muscle, but very little is known about the effect of *PLN* mutations in skeletal muscle. In this study, we investigated both histological and functional features in skeletal muscle tissue and muscle-derived myoblasts from an Italian patient carrying the *Arg14del* mutation in *PLN*. The patient has a cardiac phenotype, but he also reported lower limb fatigability, cramps and fasciculations. The evaluation of a skeletal muscle biopsy showed histological, immunohistochemical and ultrastructural alterations. In particular, we detected an increase in the number of centronucleated fibers and a reduction in the fiber cross sectional area, an alteration in p62, LC3 and VCP proteins and the formation of perinuclear aggresomes. Furthermore, the patient’s myoblasts showed a greater propensity to form aggresomes, even more marked after proteasome inhibition compared with control cells. Further genetic and functional studies are necessary to understand whether a definition of PLN myopathy, or cardiomyopathy *plus*, can be introduced for selected cases with clinical evidence of skeletal muscle involvement. Including skeletal muscle examination in the diagnostic process of *PLN*-mutated patients can help clarify this issue.

## 1. Introduction

Phospholamban (PLN) and sarcolipin (SLN) are small proteins localized in the membrane of the sarcoplasmic and endoplasmic reticula. They belong to the “regulin family”, along with myoregulin (MLN), dwarf open reading frame (DWORF), endoregulin (ELN) and another-regulin (ALN). Regulins are involved in the regulation of calcium signaling and in the activity of the sarcoplasmic reticulum Ca^+2^-ATPase pumps, SERCA1a and SERCA2a [1]. PLN is mainly expressed in cardiac, slow-twitch skeletal muscle and smooth muscle [2] and SLN in fast-twitch skeletal and cardiac muscle; both PLN and SLN play a critical role in muscle contraction [3]. In cardiac muscle, PLN is involved in the regulation of activity and the storage of calcium as a primary mediator of the β-adrenergic stimulation. In myocytes, the regulation of the SERCA2a pump by PLN depends on its phosphorylation phase. In a dephosphorylated state, PLN interacts with SERCA2a and inhibits the calcium flux; upon phosphorylation (by protein kinase A at serine 16, or by Calcium/calmodulin-dependent protein kinase II and protein kinase B at threonine 17) [4], PLN dissociates from SERCA2a ceasing its inhibitory activity. On the other hand, SLN modulates SERCA activity in skeletal muscle playing a role in muscle contraction and muscle-based thermogenesis [5].

Several human mutations have been identified in the *PLN* gene causing a cardiac disease associated with variable phenotypes including arrhythmogenic and dilated cardiomyopathy. One genetic variant found in two large Greek families harbours a stop codon for lysine 39 (Lys39stop) and causes the expression of an instable truncated protein [6]. Arg9Leu and Arg9His mutations lead to altered PLN phosphorylation by PKA [7], whereas Arg9Cys, Arg25Cys and Arg14del, in the coding region of PLN, seem to prevent phosphorylation of the PLN protein with consequent super-inhibition effects on SERCA2a activity [6,8,9]. Among these pathogenic variants, the variant c.40_42delAGA, that causes the deletion of arginine 14 (p.Arg14del) within a conserved domain of PLN, has been extensively studied. This mutation, identified for the first time in a Greek family [10], was also found in several other countries [11,12], and it is the most prevalent mutation detected in Dutch patients [13]. Clinical presentation for this mutation is severe and characterized by ventricular arrhythmias, heart failure and sudden cardiac death [14]. To date, the patho-mechanism underlying *PLN* mutations is not fully understood and a specific therapy is not yet available. It is known that this mutation causes an inhibition of SERCA2 with consequent calcium overload and cardiomyocyte damage [10,15]. Recently, it was postulated that p.Arg14del mediates the impairment of the ER/mitochondria compartment in patient-derived human induced pluripotent stem cell-cardiomyocytes, further suggesting a cytosolic Ca^+2^ scavenger as a therapeutical target [16]. The evaluation of the pathomechanisms of Arg14del was also performed in mouse models. PLN-R14 mice showed cardiac dysfunctions, extensive myocardial fibrosis and the aggregation of PLN proteins in cardiomyocytes, more severe in the homozygous mouse. This model well recapitulates features of human disease and demonstrates the inefficacy of eplerenone and metoprolol to improve cardiac function [17], whereas the early administration of a *Pln*-targeting antisense oligonucleotide showed an improvement of cardiac function and the complete elimination of PLN aggregates [18]. The p.Arg14del was demonstrated to cause electrical alteration in human-induced pluripotent stem cell-cardiomyocytes [19,20]. A more recent study evaluated the effects of p.Arg14del on the interaction between mutated PLN and PLN binding proteins, demonstrating that this mutation increases the binding of PLN to SERCA2a and HS-1-associated protein X-1 (HAX-1) with consequently aberrant calcium homeostasis and arrhythmogenesis [15]. In order to investigate the pathogenic mechanisms of p.Arg14del, Dave and colleagues recently evaluated the efficacy of in vivo genome editing in humanized mice expressing the heterozygous Arg14del mutation and demonstrated an improvement of cardiac function [21].

The histological evaluation of cardiac muscle biopsies from patients carrying the Arg14del mutation showed the peculiar presence of severe fibrosis [22,23] and perinuclear PLN-containing aggregates [24,25]. Large and dense perinuclear PLN-containing aggregates were demonstrated by immunohistochemistry in heart specimens from patients carrying the Arg14del mutation. These aggregates also showed positivity for p62 and microtubule-associated protein light chain 3 which suggests degradation by selective autophagy [24]. The immunohistochemical identification of PLN positive aggregates proved to be selective for the Arg14del mutation, thus validating the use of PLN immunostaining in cardiac muscle biopsy for the diagnosis of Arg14del-mutated cardiomyopathy [25]. Furthermore, as PLN mutated patients have a predominantly cardiac phenotype, PLN expression and function were deeply investigated in cardiac muscle, but very little is known about the effect of *PLN* mutations in skeletal muscle.

The clinical picture of our patient, characterized by lower limb fatigability, cramps and fasciculations, prompted us to also investigate their skeletal muscle tissue. To our knowledge, only one other Arg14del-mutated patient with skeletal muscle involvement (proximal limb weakness) has been reported, though not further investigated [26].

In this study, we investigated both histological and functional features in skeletal muscle tissue and muscle-derived myoblasts from an Italian patient carrying the Arg14del mutation in the *PLN* gene.

## 2. Materials and Methods

### 2.1. Clinical Data

We describe a 47-year-old-male who came to our observation and underwent skeletal muscle biopsy following a six-month history of lower limb fatigue, cramps and fasciculations. The patient was suffering from a biventricular arrhythmogenic cardiomyopathy and carried heterozygous mutations in *PLN* gene (c.40_42delAGA, p.Arg14del). Our patient had been undergoing regular cardiological evaluations for 20 years for recurrent palpitations. The surface electrocardiogram was characterized by a regular sinus rhythm interrupted by frequent ventricular extrasystoles, low-voltage QRS complexes and alterations of left ventricular repolarization. At the age of 44, he underwent cardiac magnetic resonance imaging with paramagnetic contrast medium, which documented the presence of moderate dilatation and contractile dysfunction of both ventricles, with extensive areas of epi- and intramyocardial fibrosis. Arrhythmic evaluation using serial monitoring with 12-lead Holter ECG documented persistent, polymorphic ventricular extrasystole originating from both ventricles, and some short sequences of non-sustained ventricular tachycardia. Given the clinical and instrumental findings, a diagnosis of arrhythmogenic cardiomyopathy with biventricular involvement was made and genetic screening was performed using Next Generation Sequencing method focusing on the panel of genes associated with cardiomyopathies. The genetic analysis documented the presence of the c.40_42delAGA mutation in the *PLN* gene, which encodes for phospholamban, translating into the deletion of the amino acid arginine in position 14 (p.Arg14del). Standard heart failure therapy was started, and at age 45, the patient underwent defibrillator implantation for primary prevention of sudden death, as his arrhythmic risk was deemed particularly high. Over the last few years, the patient complained of worsening asthenia and pain, mainly affecting quadricep muscles.

### 2.2. Skeletal Muscle Tissue

Skeletal muscle biopsy from patient’s left quadriceps was performed in our Neuromuscular and Rare Diseases Unit and analyzed by both light and electron microscopy. Muscle sections from four patients (average age 41 ± 4.3 years) without any detectable muscle diseases were used as normal controls (all patients had signed written informed consent before undergoing muscle biopsy). The subject gave his informed consent to both skeletal muscle biopsy and any required diagnostic tests.

#### 2.2.1. Histological and Histochemical Analysis

Tissue specimens were frozen in isopentane-cooled liquid nitrogen and processed according to standard techniques [27]. For histological analysis, 8 µm-thick cryosections were picked and processed for routine staining with Haematoxylin and Eosin (H&E), Modified Gomori Trichrome (MGT), myosin ATPase (pH 9.4-4.6-4.3), cytochrome c oxidase (COX), succinate dehydrogenase (SDH), phosphatase acid, NADH, Oil Red O and Periodic Acid Schiff (PAS). The number of centronucleated fibers and connective/adipose tissue deposition were calculated on H&E-stained sections [28], whereas ATPase-stained sections (pH 4.6) were used to determine the cross-sectional area (CSA) in type I, IIA and IIB fibers [29].

#### 2.2.2. Muscle Section Immunofluorescence Staining

Immunofluorescence staining was performed on 8 μm-thick cryostat sections using the following antibodies: anti-phospholamban-PLN (1:200, rabbit polyclonal; Invitrogen Life Technologies, Carlsbad, CA, USA), anti-MYH1 (1:100, mouse monoclonal; Abcam, Cambridge, UK), anti-p62 (1:100, rabbit polyclonal; Abcam), anti-LC3 (1:100, rabbit polyclonal; ThermoFisher, Waltham, MA, USA), anti-valosin-containing protein VCP (1:100 mouse monoclonal; ThermoFisher) and anti-caveolin-3 (1:1000, mouse monoclonal; BD Transduction, East Rutherford, NJ, USA). Slides were then incubated with the appropriate secondary antibody conjugated to Alexa 488 or Alexa 568 (1:2000, Invitrogen Life Technologies). Sections were mounted in Vectashield anti-fade mounting medium containing DAPI (Vector Laboratories, Burlingame, CA, USA). Image fields were acquired using optical microscope Leica DM4000B equipped with camera (DFC420C).

#### 2.2.3. Electron Microscopy

For ultrastructural examination, a small part of muscle sample was fixed in 2.5% glutaraldehyde (pH 7.4), post fixed in 2% osmium tetroxide and then, after dehydration in a graded series of ethanols, embedded in Epon’s resin. Finally, ultrathin sections were stained with lead citrate and uranyl acetate and examined with Zeiss EM109 transmission electron microscope.

#### 2.2.4. Western Blot

Western blot was performed on 25 μg of muscle tissue lysate. Samples were resolved on 12% polyacrylamide gels, transferred to nitrocellulose membranes and incubated with the following antibodies: p62 (1:600, rabbit polyclonal; Sigma Aldrich, St. Louis, MO, USA), PLN (1:750, rabbit polyclonal; Invitrogen), VCP (1:600 mouse monoclonal ThermoFisher) and LC3B (1:600, rabbit polyclonal; Sigma Aldrich). Actinin (1:7000, monoclonal antibody; Sigma Aldrich) and actin (rabbit polyclonal, 1:5000; Sigma Aldrich) were used as indicator of protein loaded. The membranes were incubated with rhodamine or fluorescein donkey anti-mouse or anti-rabbit secondary antibodies (LI-COR, Lincoln, NE, USA). Immunoreactive bands were visualized by ODYSSEY LI-COR Model 2800.

#### 2.2.5. Primary Skeletal Muscle Cell Cultures

Primary cell cultures were derived directly from biopsied material. Muscle biopsy was cleaned of the connective component and minced into small fragments. These were cultured in Skeletal Muscle Growth Medium completed with Growth Medium Supplement (PromoCell, Heidelberg, Germany) in six-well plates. The medium was changed twice weekly and the cultures examined by inverted-phase microscopy. When they reached 80% confluence they were dissociated enzymatically with trypsin-EDTA (Sigma) and seeded for immediate propagation, or frozen in medium containing 10% DMSO (Sigma). Primary isolated myoblasts were analyzed for mycoplasma contamination with MycoAlert Plus Mycoplasma detection kit (Lonza Group, Basel, Switzerland).

The mitochondria morphology in patient’s and controls’ myoblasts was evaluated with MitoTracker Deep Red (ThermoFisher). Myoblasts were incubated with MitoTracker at 100 nM for 30 min at 37 °C and 5%CO_2_. Then, cells were washed with PBS and mounted in Vectashield anti-fade mounting medium containing DAPI (Figure S1 Supplementary).

#### 2.2.6. Myoblasts PLN Immunofluorescence Staining

Immunofluorescence staining was performed on primary myoblasts isolated from control and patient biopsies and seeded on glass coverslips. At 70–80% of confluence, cells were fixed with 4% of paraformaldehyde for 30 min at RT. After PBS washing, cells were permeabilized with 0.1% TritonX-100 in PBS for 15 min. The unspecific sites were blocked with 1% BSA in PBS for 45 min. Cells were incubated with the primary antibody anti-PLN (1:200, rabbit polyclonal; Invitrogen) for 2 h at RT and then incubated with the goat anti-rabbit secondary antibody conjugated with Alexa 488 (1:1000) for 1 h. Slides were mounted in Vectashield anti-fade mounting medium containing DAPI.

#### 2.2.7. In Vitro Detection of Aggresomes

The experiments to detect the formation of aggresomes were performed using ProteoStat^®^ Aggresome Detection Kit (EnzoChem, Farmingdale, NY, USA) according to manufacturer’s instructions. In brief, control and patient primary myoblasts were seeded on coverslips. At 80% of confluence, cells were treated with the proteasome inhibitor MG-132 at 2 µM, or with DMSO as control vehicle, for 18 h at 37 °C and 5% of CO_2_. The cells were subsequently washed with Phosphate Buffer Solution (PBS) and fixed with 4% formaldehyde for 30 min at room temperature (RT). After washing with PBS, cells were permeabilized with 0.5% Triton X-100 and 3 mM EDTA in PBS for 30 min on ice. After washing with PBS, Dual Dye ProteoStat dye (also containing Hoechst for nuclei counterstaining) was added to the cells for 30 min at RT. Cells were washed with PBS and coverslips were mounted in anti-fade mounting medium (Vector Laboratories). Image fields were acquired using optical microscope Leica DM4000B equipped with camera (DFC420C) at 40×.

#### 2.2.8. Co-Localization of Aggresomes and Autophagy Markers on Myoblasts

For antibody co-localization studies, cells were treated with the proteasome inhibitor MG-132 at 2 µM, for 18 h at 37 °C and 5% of CO_2_, then fixed and permeabilized using the above protocol. Then, cells were incubated in blocking buffer (BSA 1% in PBS) for 30 min at RT and incubated with one of the following primary antibodies: anti-PLN (1:200), anti-p62 (1:100), anti-LC3 (1:100) for 2 h at RT, followed by incubation with the secondary antibody goat anti-rabbit conjugated to Alexa 488 (1:1000, ThermoFisher). Cells were then washed in 0.1% Triton X-100 in PBS (for 15 min at RT) and exposed to ProteoStat for 30 min at RT. Cells were washed with PBS and coverslips were mounted in anti-fade mounting medium (Vector Laboratories).

### 2.3. Statistical Analysis

Statistical analysis on histological data was performed using GraphPad Prism 5 software (GraphPad Software, LaJolla, CA, USA). Significant levels were set as *p* ≤ 0.001 (**). Preliminary ANOVA test was applied to compare control data sets. The data are expressed as mean ± standard error of the mean (SEM). An unpaired two-tailed *t*-test was applied to compare data from controls and patient. Polynomial trendline evaluation for fiber type distribution was performed using Excel software.

## 3. Results

### 3.1. Muscle Biopsy: Histology and Histochemistry Modifications and Fiber CSA Reduction

To evaluate possible morphological or enzymatic alterations in a skeletal muscle biopsy from the patient compared with control muscle.

**Figure 1:**
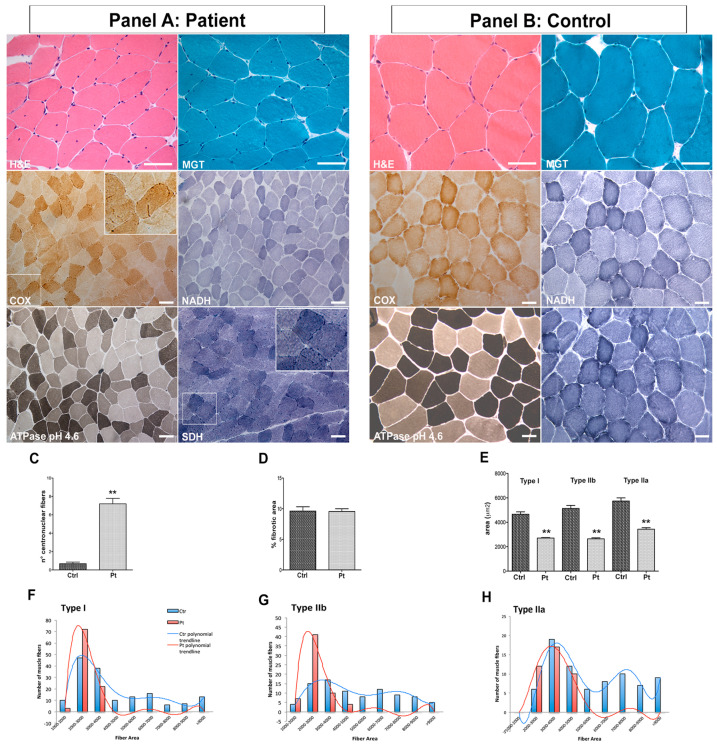
Skeletal muscle biopsy. Histological staining (H&E and MGT) and enzymatic activities (COX, NADH, ATPase pH 4.6 and SDH) in patient (Panel **A**) and in control (Panel **B**). H&E and MGT showed fiber size variability and centronucleated fibers in patient compared with the age-matched control. Scale bar 50 μm. The inset shows a magnification of COX and SDH staining in patient muscle section. Histogram for the number of centronucleated fibers shows significant increase in centronucleated fibers in patient compared with controls (n° fibers counted = 678 in patient and 912 in controls; n° controls = 4; *p* < 0.0001) (**C**); fibrosis quantification showed no difference between patient and controls in connective and adipose tissue deposition (n = 4 controls; *p* = 0.9407) (**D**). CSA evaluation for type I (n° fibers counted = 99 for patient and 173 for controls; *p* < 0.0001), type IIb (n° fibers counted = 65 for patient and 91 for controls; *p* < 0.0001) and type IIa fibers (I) (n° fibers counted = 42 for patient and 81 for controls; *p* < 0.0001; n° controls = 4) showed significant reduction in CSA of all the three types of fibers (**E**); ** *p* < 0.0001. Fiber size distribution showed a difference in polynomial trendlines between patient and controls for all the three types of fibers (**F**–**H**).

H&E and MGT stains showed homogeneously shaped fibers with physiological size variability except for the presence of scattered round-shaped hypotrophic fibers (Figure 1 Panel A). The percentage of centronucleated fibers was significantly increased (15.929%) compared with the controls (1.864%, *p* < 0.0001) (Figure 1C). No fiber splittings, necroses or cellular infiltrates were observed. Both endomysial and perimysial connective tissue were normal. Fibrosis quantification showed similar results in both the patient (9.605% ± 2.571% area; *p* = 0.9407) and the controls (9.544% ± 2.259 % area) (Figure 1D). The enzymatic activities for COX, NADH, SDH (Figure 1 Panel A) and acid phosphatase were normal. No changes in glycogen and lipid content were observed. The COX-positive fibers were counted in both patient and controls and distinguished in strongly positive and lightly positive fibers. The patient presented 29.69% of strongly positive fibers compared with 33.09% of the controls (*p* = 0.0934) and 70.37% of lightly positive fibers compared with 66.90% of the controls (*p* = 0.0029). CSA quantification showed a significant area reduction in all the three fiber types analyzed: the mean CSA for type I fibers was 2707 µm^2^ ± 38 µm^2^ for the patient and 4654 µm^2^ ± 202 µm^2^ for the controls (*p* < 0.0001), for type IIb fibers it was 2640 µm^2^ ± 88.86 µm^2^ for the patient and 5130 µm^2^ ± 245 µm^2^ (*p* < 0.0001) for the controls and for type IIa fibers, it was 3433 µm^2^ ± 125 µm^2^ for the patient and 5738 µm^2^ ± 258 µm^2^ for the controls (*p* < 0.0001) (Figure 1E). The percentage distribution for the three fiber types showed no significant differences in patient vs. controls: 48.27% vs. 50.43% for type I fibers; 31.52% vs. 26.23% for type IIa fibers and 20.19% vs. 23.32% for type IIb fibers. The mean of the total CSA was 2834 µm^2^ ± 47.07 µm^2^ for the patient and 5034 µm^2^ ± 136.3 µm^2^ for the control (*p* < 0.0001). As for fiber caliber distribution, we observed different polynomial trendlines between the patient and the controls for all the three types of fibers. In more detail, the patient predominantly presented fibers of a small caliber (from 1000 to 5000 μm^2^), whereas fibers of a greater caliber were evident in the controls (up to over 9000 μm^2^) (Figure 1F–H). These results showed a generalized CSA reduction in the patient’s muscle regardless of the type of fiber evaluated. This condition likely results from both an altered cardiac function, which lowers tissue oxygenation of all fiber types, and from muscle disuse.

### 3.2. PLN Expression in Muscle Biopsy and Primary Myoblasts and Immunofluorescence Staining of Autophagic Markers in Muscle Tissue: Comparison between Patient and Controls

To evaluate the expression level of PLN and the expression levels of autophagic markers in muscle tissue.

**Figure 2:**
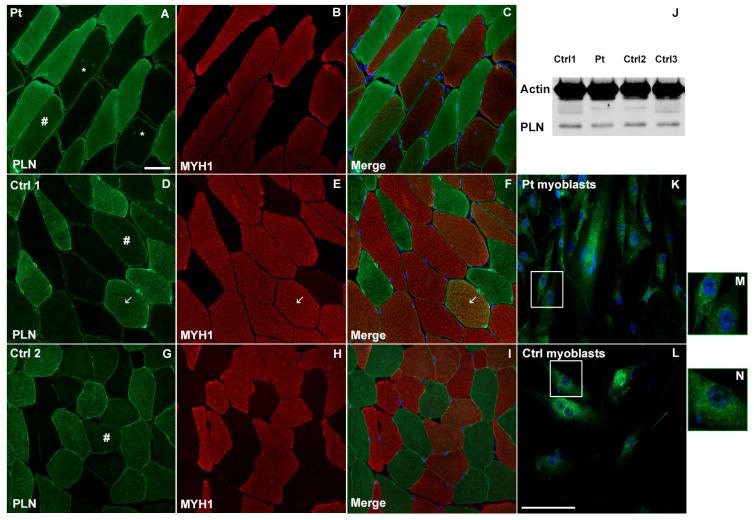
Immunofluorescence of PLN on muscle sections from patient (**A**) and controls (**D**–**G**), MYH1 staining for type II fibers in patient (**B**) and controls (**E**,**H**) and merge images for patient (**C**) and controls (**F**,**I**). Asterisks indicate PLN positivity in perinuclear region in patient’s centronucleated fibers. Hashtags and arrows indicate fibers with different “intermediate” phenotypes. Scale bar 50 μm. Western Blot for PLN in muscle sections from patient and controls (**J**). Immunofluorescence for PLN in primary myoblasts from patient (**K**) and control (**L**). Magnification box of perinuclear PLN staining in patient (**M**) and control (**N**) myoblasts. Nuclei were counterstained with DAPI. Scale bar 50 μm.

PLN staining showed a marked cytoplasm and membrane positivity in slow-twitch skeletal type I fibers in both patient and control muscle sections, as shown by the double fluorescent reaction for PLN and myosin heavy chain 1 (MYH1) staining of type II fibers. PLN positivity was homogeneously distributed throughout the cytoplasm and around the nuclei of type I fibers, in both the patient (Figure 2A) and the control muscles (Figure 2D–G). PLN expression was also observed at the membrane level in type II fibers, whereas perinuclear positivity for PLN was detected around the central nuclei in type II fibers (Figure 2A, indicated with asterisks *). In both the patient and control muscle sections, some fibers with a diffuse cytoplasmic intermediate PLN fluorescent signal between type I and type II fibers were observed (Figure 2A,D,G, indicated with hashtag #) along with a type II fiber with strong PLN positivity (Figure 2D–F, indicated with arrow). A Western blot experiment showed similar PLN bands for both the patient and the controls (Figure 2J). Immunostaining with a PLN antibody in the primary undifferentiated myoblasts also confirmed the presence of the protein in muscle cells, showing a perinuclear punctate positivity with a similar pattern in both the patient (Figure 2K) and the control cells (Figure 2L).

**Figure 3:**
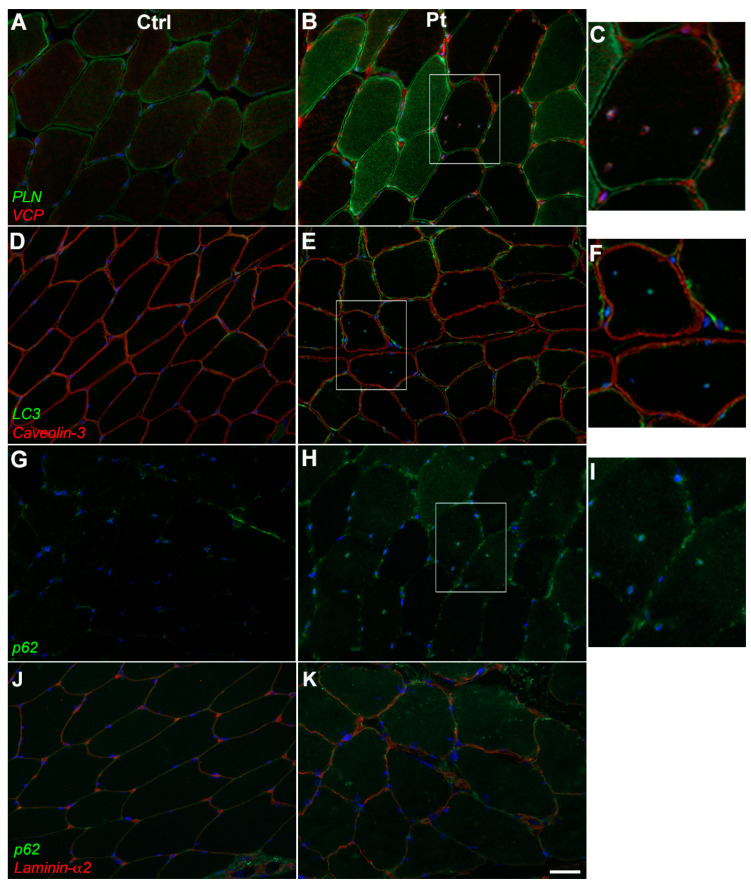
Immunofluorescence of autophagic markers in control and patient muscle sections. In patient muscle, VCP signal specifically localizes at interfiber level and in proximity of nuclei (**A**,**B**), LC3 signal is increased in patient at both sarcolemmal and at perinuclear level (**D**,**E**). Caveolin-3 was used to detect plasma membranes. P62 staining alone (**G**,**H**) and in double immunofluorescence with laminin-α2 (**J,K**) appeared increased in the patient with a punctate distribution along the sarcolemma of some fibers as well as in the perinuclear area. Magnification of centronucleated fibers in patient muscle sections (**C**,**F**,**I**). Nuclei were counterstained with DAPI. Scale bar 50 μm.

These results confirm the previously reported observations of PLN expression and localization during normal myocyte differentiation [30] as well as data obtained in cell lines transfected for endogenous and Arg14del-mutated PLN forms [10]. Immunofluorescence studies were also performed to evaluate the expression of some autophagic markers, namely Valosin-containing protein (VCP), LC3 and p62/SQSTM1. P62 is an adaptor protein involved in the sequestration, aggregation and degradation of misfolded proteins; LC3 is involved in the recruitment of autophagic cargoes into autophagosomes [24,25], while VCP is involved in the identification of ubiquitinated proteins during the autophagic process and in aggresome formation [31]. In the patient’s muscle, VCP especially localized at an interfiber level and in the proximity of nuclei, more evidently so in fibers with centralized nuclei where VCP partially co-localized with PLN (Figure 3B,C). In muscle sections from healthy controls, the VCP signal at both perinuclear and interfiber levels appeared slightly less marked compared with the patient’s (Figure 3A). Similar to VCP, in the patient’s muscle, an LC3 signal was detected at the perinuclear level. Moreover, scattered LC3 positivity was observed along the sarcoplasma of some fibers (Figure 3E,F). The positivity for LC3 in the patient muscle was moderately stronger than in the healthy controls (Figure 3D). A P62 fluorescent signal appeared more diffusely increased in the patient muscle compared with the healthy controls, a punctate staining being evident at the sarcolemma of some fibers as well as in the perinuclear area and in the cytoplasm of some fibers (Figure 3H–K).

### 3.3. Western Blot on Muscle Tissue and of Autophagic Markers in Primary Myoblasts Showed Slight Increase in p62

To analyze autophagic marker expression in muscle biopsy.

**Figure 4:**
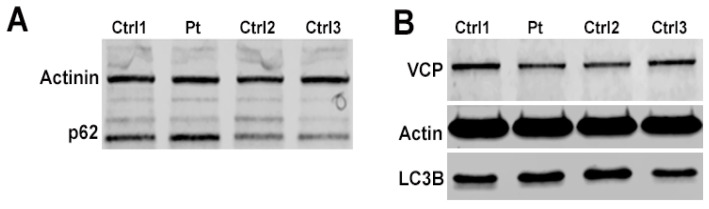
Western Blot for autophagic markers p62 (**A**), VCP and LC3B in skeletal muscle from patient and three controls (**B**).

Qualitative observation of Western blot bands for autophagic markers showed a slight increase in the p62 band for the patient compared with the controls (Figure 4A), whereas the VCP signal was more difficult to compare given the variability observed among the controls. LC3B immunoblot showed similar cytosolic LC3B-I bands for both the patient and the controls, whereas the autophagosome membrane LC3B-II isoform was not detected in all samples (Figure 4B).

### 3.4. Electron Microscopy on Patient Muscle Tissue Revealed the Presence of Aggresomes

To identify possible ultrastructural alterations in muscle biopsy as a consequence of Arg14del PLN mutation.

**Figure 5:**
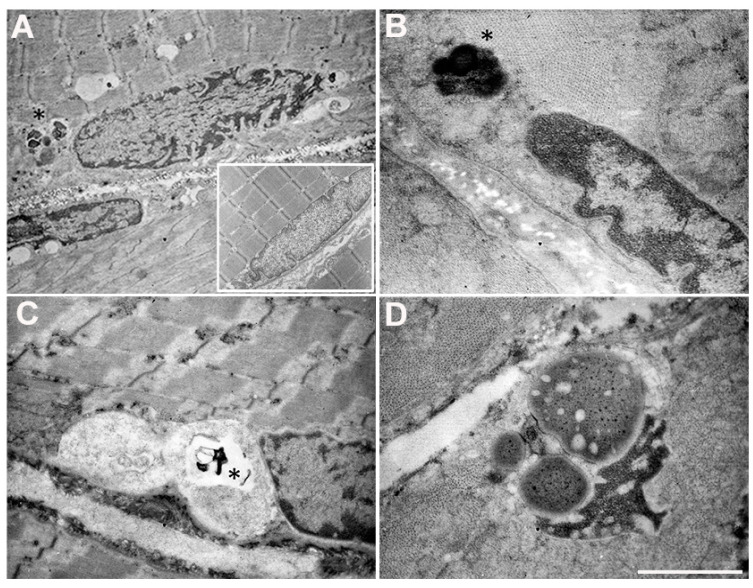
Ultrastructural examination showing the main alterations in the nuclear and perinuclear regions. Invaginations of nuclear membrane (**A**). Electrondense structures localized close to the nucleus (**A**,**B**, asterisks). Vacuoles containing finely granular material and electrondense structures (**C**, asterisk). Large lipofuscines close to an affected nucleus with highly condensed and compacted chromatin (**D**). The inset (image **A**) shows a normal nucleus. Scale Bar: (**A**): 2.27 µm, (**B**): 0.5 µm, (**C**): 1.43 µm, (**D**): 0.84 µm.

A careful ultrastructural examination of the nuclear regions showed several nuclear alterations; the most significant morphological changes are reported in Figure 5. Electrondense structures, small to moderate in size (asterisks), were frequently observed close to the nuclei (Figure 5A,B), often associated with invaginations of the nuclear membrane (Figure 5A). These electrondense structures were sometimes seen inside vacuoles, together with finely granular material (Figure 5C). The nuclear morphology was frequently altered by the presence of large lipofuscines, the nuclear chromatin being highly condensed and compacted into half-moon shapes (Figure 5D). These ultrastructural observations were suggestive of the formation of aggresomes or aggresome-like structures, similar to the structures observed in the cardiac muscle of Arg14del-mutated patients [24].

**Figure 6:**
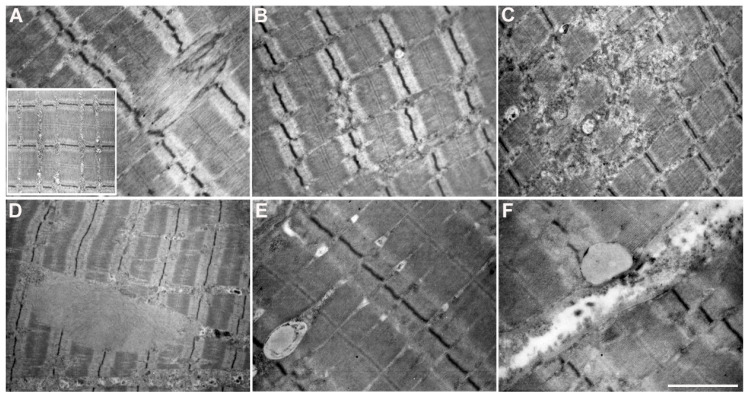
Ultrastructural examination of the sarcomeric region in patient skeletal muscle. Disruption of the Z-bands with longitudinal streaming (**A**) or absence of Z-line (**B**). Focal area of myofibrillar disorganization (**C**). Inclusion of thin filaments in the sarcoplasm (**D**). Lipid droplets at intracytoplasmic (**E**) and at subsarcolemmal level (**F**). The inset (in image (**A**)) represents a normal sarcomeric region. Scale bar: (**A**–**C**): 1.43 µm, (**D**): 2.27 µm, (**E**,**F**): 0.84 µm.

In addition, we observed further structural changes in a fair number of fibers. The sarcomeric structure was partially disrupted in several muscle fibers where we observed either streaming (Figure 6A) or absence (Figure 6B) of Z line. In some cases, the sarcomeric alterations were of remarkable size and involved several sarcomeres, thus causing focal areas of myofibrillar disorganization (Figure 6C). Thin filamentous inclusions are shown in Figure 6D. In addition, lipid droplets were observed in a fair number of fibers, at both intracytoplasmic (Figure 6E) and subsarcolemmal level (Figure 6F). These observations are in line with what has been reported in Arg14del PLN-mutated mouse hearts exhibiting alterations in Z-disc formation or organization [21,32], and by Pei who observed the accumulation of lipid droplets and altered mitochondrial integrity in the heart of mutated patients [33].

### 3.5. Aggresomes Formation in Myoblasts Showed the Presence of Numerous Perinuclear Aggregates after Proteasome Inhibition

To evaluate the tendency to form aggresomes in primary myoblasts isolated from patient skeletal muscle in basal condition and after proteasome inhibition.

**Figure 7:**
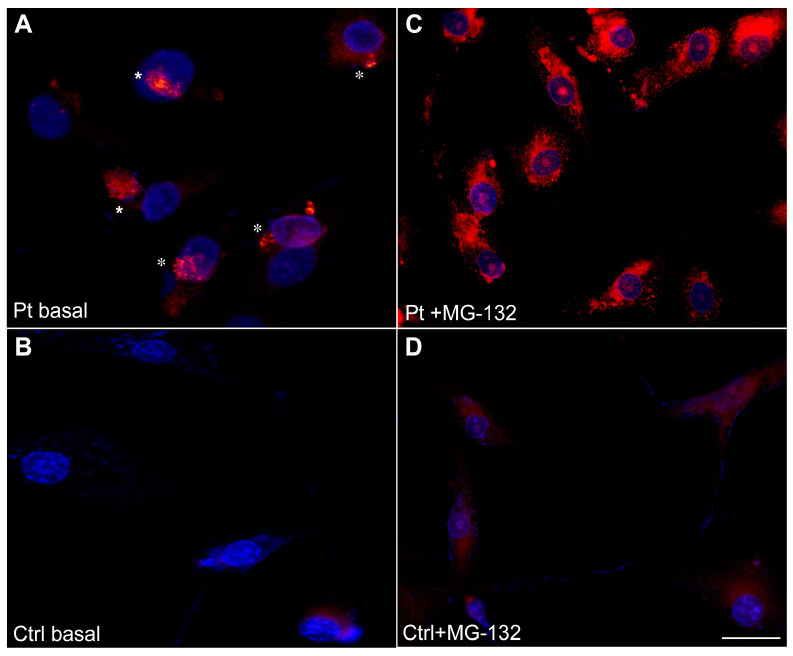
Detection of aggresomes in patient’s myoblasts. In basal conditions, numerous perinuclear aggregates, appearing as single ProteoStat-positive spheres, were detected in patient myoblasts (**A**, asterisks). No aggregates were detected in control cells (**B**). After treatment with the proteasome inhibitor MG-132 for 18 hrs, the ProteoStat positivity in patient’s myoblasts was more markedly distributed in widespread aggregates around the nuclei and in a diffuse cytoplasmic pattern (**C**), whereas only a very faint cytoplasmic signal was detected in control myoblasts (**D**). Nuclei were counterstaining with Hoechst. Scale bar 50 µm.

The observations obtained from immunofluorescent and ultrastructural evaluations in the patient’s muscle biopsy suggest the possible formation of aggresomes or aggresome-like structures in Arg14del-mutated skeletal muscle similar to what occurs in cardiac muscle even if the presence of PLN-aggregates in the skeletal muscle biopsy was less diffused than that observed in cardiac muscle biopsies and seemed not to involve PLN. To investigate these aspects, we performed experiments to evaluate the propensity of the formation of aggresomes in primary myoblasts isolated from the patient’s skeletal muscle. The experiments on primary undifferentiated myoblasts from the patient showed, in still basal conditions, numerous perinuclear aggregates, appearing as single spheres, positive to ProteoStat dye (Figure 7A). No aggregates were detected in the control cells (Figure 7B). After treatment with the proteasome inhibitor, MG-132, the fluorescent signal in the patient’s myoblasts was more markedly distributed in widespread aggregates around the nuclei and in a diffuse cytoplasmic pattern (Figure 7C) compared with control cells in which only a very faint cytoplasmic signal was detected. These results suggest a greater responsiveness to MG-132 of the patient’s myoblasts compared with the control cells (Figure 7D).

### 3.6. Evidence of Co-Localization of Aggresomes and Autophagy Markers on Myoblasts

To evaluate the co-localization of aggresomes and autophagic markers in basal conditions in primary patient’s myoblasts.

**Figure 8:**
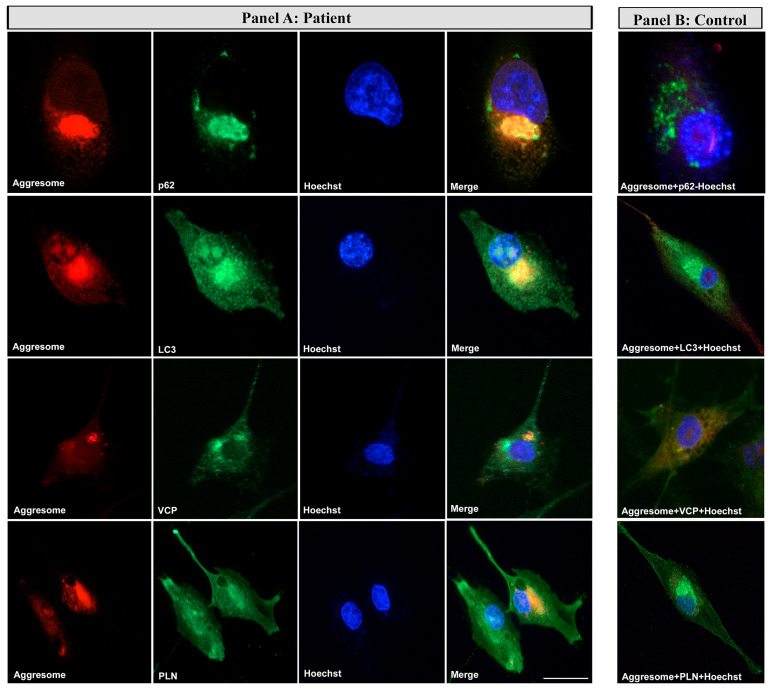
(Panel **A**): co-localization of aggresomes and autophagic markers in patient’s myoblasts. The images show ProteoStat aggresome detection (red), autophagic markers (green), nuclei (blue) and merge in patient’s myoblasts. PLN staining shows a partial co-localization with aggresomes; (Panel **B**): merge images for control myoblasts. Scale bar 50 µm.

The patient’s myoblasts were analyzed for autophagic markers in basal conditions. The patient’s myoblasts showed positivity to p62, LC3 and VCP associated with aggresomes (Figure 8 Panel A). P62 and LC3 positivity co-localized with a ProteoStat signal associated with aggresomes, a pattern similar to the one reported in cardiac tissue from Arg14del-mutated patients. Furthermore, VCP, involved in the recognition of unfolded proteins, co-localized with aggresomes at the perinuclear region (Figure 8 Panel A) in the patient’s myoblasts. Furthermore, the staining for PLN in the patient’s myoblasts also showed a partial co-localization with aggresomes (Figure 8 Panel A).

## 4. Discussion

Mutations in the *PLN* gene are responsible for a severe cardiac phenotype with an arrhythmogenic, as well as dilated, cardiomyopathy associated with a severe prognosis and high mortality. As described by Hof and coll., patients carrying the heterozygous mutations Arg14del are characterized by older age of onset, low-voltage electrocardiograms and a high frequency of ventricular arrhythmias. Additionally, these patients have a poor prognosis and early-onset heart failure [13]. Because PLN mutated patients have a predominantly cardiac phenotype, PLN expression and function have been deeply investigated in cardiac muscle, but very little is known about the effect of PLN mutations in skeletal muscle. In a study conducted to determine the phenotypic spectrum associated with *PLN* mutations, DeWitt and coll. described an Arg14del-mutated proband complaining of skeletal muscle weakness with a limb girdle distribution. As PLN is expressed in slow skeletal muscle, any alteration could also be associated with a skeletal muscle phenotype [26]. Indeed, our patient reported lower limb fatigability, cramps and fasciculations which were highly suggestive of skeletal muscle involvement and prompted us to investigate his skeletal muscle tissue.

The histological evaluation of the patient’s muscle biopsy showed a significant increase in centronucleated fibers and a generalized marked reduction in fiber CSA, but no increase in connective or adipose tissue. Immunofluorescence studies on muscle biopsy showed a PLN signal similar to controls in both the membranes and cytoplasm of type I fibers. Membrane positivity was also detected in type II fibers in all samples. In some fibers, a cytoplasmic PLN signal was less evident than in type I fibers, which may indicate the presence of fibers with an intermediate phenotype [34]. The presence of PLN in both type I and II fibers was previously reported in human vastus lateralis fibers by single fiber Western blot analysis [35].

In the patient’s muscle sections, perinuclear positivity was also observed in type II fibers with nuclear centralizations and in primary myoblasts from both the patient and controls.

In cardiac muscle, te Rijdt and colleagues thoroughly described the formation of dense, globular PLN-positive perinuclear aggregates that are degraded via autophagosomes and lysosomes and that appeared as aggresomes at an ultrastructural examination. These aggresomes positively immunostained to p62- and LC3-, that co-localize with PLN [24,25]. The formation of these PLN-positive aggresomes was also demonstrated to be specific for Arg14del-mutated cardiac tissue [25]. In the skeletal muscle from our patient, perinuclear positivity for p62 and LC3 was observed. Furthermore, we also detected VCP positivity localized at the interfiber level and in proximity of the nuclei, more evidently so in fibers with centralized nuclei where VCP had partially co-localized with PLN. However, differently from cardiac muscle, PLN-positive aggregates were not evident in the skeletal muscle after immunofluorescence investigation. The expression levels of autophagic markers on skeletal muscle tissue showed only a slight increase in p62, but no clear differences in both VCP and LC3B protein levels. An ultrastructural examination of the patient’s muscle tissue showed the presence of perinuclear electrondense structures, suggesting the formation of aggresomes or aggresome-like structures, as observed in Arg14del PLN-mutated cardiac tissue. However, differing from what observed in cardiac tissue, these formations did not seem to contain PLN. To further investigate the formation of aggresomes, we used an in vitro model more dynamic than muscle sections, based on primary myoblasts derived from the muscle biopsy of our patient. In cellular basal growth conditions, this model already showed the greater propensity of patient’s myoblasts to form aggresomes compared with the control cells, and this tendency became even more marked after proteasome inhibition. Immunocitochemistry on these cells confirmed the signal positivity for p62, LC3 and VCP associated with aggresomes. In the patient’s myoblasts, an association of the fluorescent signal for PLN with aggresomes was also observed. The formation of aggresomes, initially described by Johnston JA and coll. (1998) [36], is a dynamic process and can be considered a cellular response for mitigating proteotoxic stress that occurs when the production of misfolded/mutated proteins exceeds the capacity of the proteasome. The process of aggresome formation reduces the cytotoxic effects of scattered cellular protein aggregates, even if their long-term persistence can be detrimental for cell viability [37]. Aggresomes, constantly evolving structures capable of recruiting ubiquitination enzymes, chaperones and proteasome components [38] have been found in other pathological conditions characterized by the formation of protein aggregates, namely in Alzheimer’s, Parkinson’s and Huntington’s diseases; amyloid and prion disease [38,39]; in hereditary reducing-body myopathy [40] and in a sarcopenic patients with rigid spine syndrome and a mutation in the FHL1 gene [41].

We made other relevant observations from the evaluation of CSA in skeletal muscle from our patient. Indeed, we found a significant area reduction in all the three types of analyzed fibers compared with controls. The distribution of these fibers by dimension indicates that the patient mainly presented only small area fibers compared with controls, as demonstrated by the difference in the polynomial trendlines. The observed reduction of CSA was not due to a replacement of muscle fibers with connective or adipose tissue as demonstrated by the detection of quite similar fibrosis values in both the patient and controls. This generalized, not fiber-type related, CSA reduction likely results from both an altered cardiac function, which lowers tissue oxygenation of all fiber types, and from muscle disuse. The basis for the selectivity of fiber-type muscle atrophy remains an important and unresolved issue, different signaling pathways possibly being involved. In general, disuse-related skeletal muscle atrophy, that typically occurs during denervation and/or immobilization, is primarily related to oxidative fibers, whereas nutrient-related atrophy, i.e., cancer/aging cachexia, sepsis, chronic heart failure and diabetes, is more directed to glycolytic fiber wasting [42]. Patients with chronic heart failure show decreased muscle bulk compared with healthy humans. The cross-sectional area of the lower extremity musculature in heart failure was reported to be significantly smaller than that in healthy humans, and, importantly, it is correlated with a decreased peak oxygen consumption [43,44]. This CSA reduction could therefore be the result of an altered proteostasis in the patient’s skeletal muscle. An impaired proteostasis usually occurs during aging and it is linked to structural and functional changes that ultimately lead to atrophy [45]. Recently, multiple isogenic IPS cell lines carrying the mutation Arg14del were analyzed by single cell RNA-sequencing by Feyen and coll. (2021) [46]. This approach revealed that Arg14del mutation induces the activation of the unfolded protein response (UPR). This pathway, together with the autophagic-lysosomal system, plays an important role in the maintenance of proteostasis [46].

Skeletal muscle contains a large endoplasmic reticulum (ER) that contributes to the regulation of proteostasis and calcium homeostasis. The persistence of misfolded or unfolded proteins, as in case of certain genetic mutations, causes ER stress and, consequently, the activation of UPR [47]. UPR pathway activation is a complex process mediated by PERK (RNA-dependent Protein kinase-like ER eukaryotic translation initiation factor 2 alpha kinase), 1RE1 (inositol-requiring protein) and ATF6 (activating transcription factor 6) [48]. ER stress and the UPR pathway are activated and involved in the physiology of skeletal muscle, i.e., in maintaining the pool of satellite cells in adult muscle, in adaptation to exercise and in the regulation of skeletal muscle mass; their exact role in the regulation of skeletal muscle mass remains poorly understood [49]. The ultrastructural analysis of Arg14del hiPSC-cardiomyocytes showed dilation of the ER and a large lipid droplet deposition in close association with mitochondria, whereas the ultrastructural analysis of the skeletal muscle of our patient did not show alteration in the ER lumen and only displayed limited deposition of lipid droplets. UPR activation and protein aggregation were also found in protein folding diseases such as Alzheimer’s, Parkinson’s and amyotrophic lateral sclerosis [47]. Furthermore, ubiquitin/proteasome and/or the autophagy/lysosomal pathways have been found altered in different forms of skeletal muscle atrophy/dysfunction [50,51,52].

In vitro experiments of proteasome inhibition showed the great propensity of patient’s myoblasts to massively form aggresomes suggesting more an involvement of the proteasome degradation pathway than an autophagic activation. Even the exact role of aggresomes or aggresome-like structures in muscle fibers still needs to be investigated, their presence, as well as their induction in cultured human patient’s myoblasts by proteasome inhibition, further supporting the prominent role of the UPR pathway. Furthermore, ER stress with UPR activation influenced both transcriptional and translational programs inducing a decrease in protein synthesis [53]. These observations suggest that the involvement of an altered UPR process and the appearance of aggresomes in muscle fibers can be related to fiber atrophy and consequent muscle weakness even if more studies need to be conducted on both skeletal muscle and in in vitro models.

## 5. Conclusions

Our findings indicate that *PLN* gene mutations can affect skeletal muscle tissue in addition to the primary cardiac involvement. Since general weakness is a common feature in patients affected with dilatative heart failure, it can be hypothesized that, at least in patients with dilatative cardiopathy due to *PLN* gene mutations, part of this weakness is a direct consequence of muscle involvement. Further genetic and functional studies are necessary to understand whether a definition of PLN myopathy, or cardiomyopathy *plus*, can be introduced for selected cases with clinical evidence of skeletal muscle involvement. Including skeletal muscle examination in the diagnostic process of PNL-mutated patients can help clarify this issue.

## Data Availability

The data for this article are not publicly available to ensure patient anonymity. Requests to access the data should be directed to the corresponding author.

## Supplementary Materials

The following supporting information can be downloaded at: https://www.mdpi.com/article/10.3390/cells12101405/s1. Figure S1: MitoTracker incubation showed a similar mitochondrial network in both control (A) and patient (B) myoblasts. Nuclei were counterstained with DAPI. Scale bar 50 μm. Figure S2: original uncropped Western Blot images.
