# Supplementary material for: Characterization of Skeletal Muscle Biopsy and Derived Myoblasts in a Patient Carrying Arg14del Mutation in Phospholamban Gene
Source: Cells. 2023 May 17;12(10):1405. doi: 10.3390/cells12101405 (PMC10216566; doi:10.3390/cells12101405)
Supplement: Supplementary file 1 [file cells-12-01405-s001.zip › cells-2317337-supplementary.pdf]

Supplementary Information

Figure S1 Supplementary

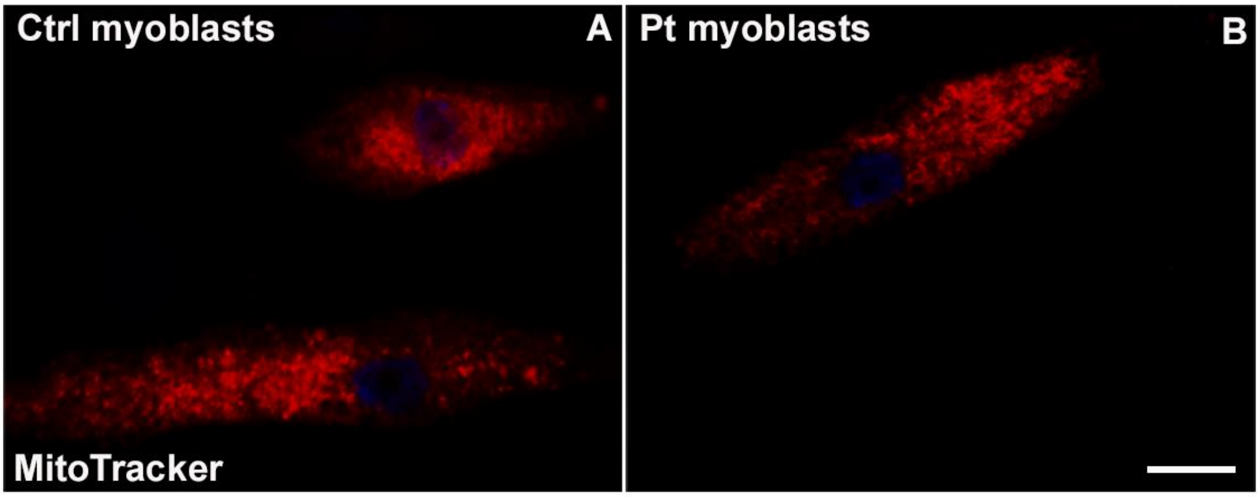

Figure S1: MitoTracker incubation showed a similar mitochondrial network in both control (A) and patient (B) myoblasts. Nuclei were counterstained with DAPI. Scale bar 50 μm.

Figure S2 Supplementary

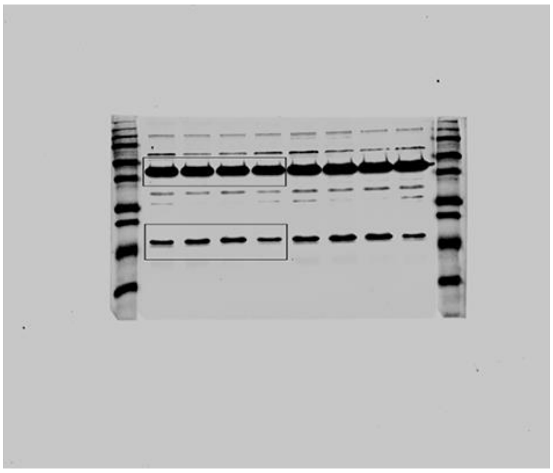

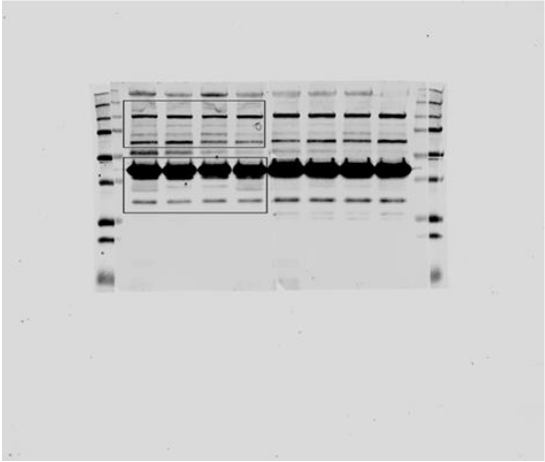

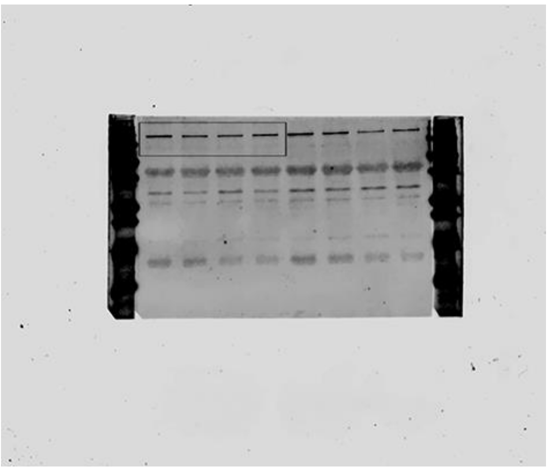

Figure S2: original uncropped Western Blot images.
